# Actinide Nanoparticles: Revising Early Developments and Recent Insights from High‐Energy‐Resolution Fluorescence‐Detected X‐Ray Absorption Near Edge Structure and Synchrotron Techniques

**DOI:** 10.1002/smsc.202500312

**Published:** 2025-11-28

**Authors:** Lucia Amidani, Clara Lisa Silva, Stephan Weiss, Kristina O. Kvashnina

**Affiliations:** ^1^ The Rossendorf Beamline at ESRF The European Synchrotron CS40220 38043 Grenoble Cedex 9 France; ^2^ Institute of Resource Ecology Helmholtz Zentrum Dresden Rossendorf 01328 Dresden Germany

**Keywords:** actinides, high‐energy‐resolution fluorescence‐detected X‐rays absorption near edge structure, nanoparticles, synchrotron techniques, X‐ray spectroscopy

## Abstract

Actinide nanoparticles (NPs) are widely recognized for their role as a potentially highly mobile form of radioactive contaminants in the environment. In recent years, research has increasingly focused on elucidating their formation mechanisms, atomic structure, and physicochemical properties. The application of synchrotron radiation techniques is central to the detailed characterization of their atomic structure and oxidation state. This review retraces the evolution of actinide NPs research and highlights recent achievements enabled by high‐energy‐resolution fluorescence‐detected X‐ray absorption near edge structure, used in correlation with complementary synchrotron‐based methods.

## Graphical abstract

Actinide nanoparticles are relevant to nuclear waste storage, environmental safety, advanced nuclear fuel engineering, and fundamental science. Herein, this review traces the early developments of the field and the recent progress enabled by the use of high‐energy‐resolution fluorescence‐detected X‐ray absorption near edge structure in synergy with other synchrotron techniques.

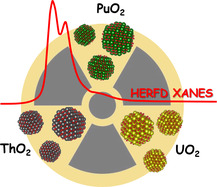

## 1 Introduction

We are nowadays familiar with the nanoworld. Often, scaling down a system to the nanometer range, along one or more spatial dimensions, introduces surprising physics and new properties, as well as revealing the role of surface atoms in the chemico‐physical processes.^[^ ^1^, ^2^ ^]^ The universe of the nano is explored in almost all fields of science. However, in this exciting exploration of the ultrasmall, the field of actinide‐based materials still lags far behind systems based on stable elements.

There are several reasons behind this delay. The main one is the complexity of handling actinides and their radiological risk. Working with actinides requires certified laboratories, and transferring actinide samples outside the laboratories where they have been synthesized implies specific safety procedures to prevent contamination. As a result, any experiment aiming at actinide characterization is complicated by their radiological risk and toxicity, and this is especially true for transuranic elements. This simple fact limits the number of groups and the experimental work performed on these elements at all levels. Additionally, it is useful to recall that most of the actinides were only discovered after 1940, making actinide science a very young field compared to the knowledge acquired on the chemistry and physics of materials based on stable elements.^[^ ^3^ ^]^

It would, however, be wrong to assume that this delay in the development of actinide nanoscience has been motivated by the lack of scientific interest. On the contrary, actinide‐bearing colloidal nanoparticles (NPs) were identified as a highly mobile form of radionuclide transport in the environment already in the early ’90s.^[^ ^4^, ^5^, ^6^ ^]^ It follows that for safe nuclear waste management, the scenario of nanoparticle formation has to be considered to control and prevent the release of radioactive pollutants in the environment. In the nuclear energy field, the possibility to change bulk properties through nanosizing is of high interest in order to improve thermal conductivity, radiation resistance, and fission product retention in view of optimizing nuclear fuels.^[^ ^7^, ^8^ ^]^ Actinides are also investigated for their catalytic properties,^[^ ^9^, ^10^, ^11^ ^]^ and recently UO_2_ NPs have been proposed for efficient gas sensing.^[^ ^12^ ^]^

The scientific relevance of actinides is not limited to nuclear safety and technology. Actinides are of the utmost importance for fundamental science.^[^ ^13^ ^]^ They sit at the bottom of the periodic table and are probably the least understood among the chemical series. They were assimilated with lanthanides for a long time before evidence of their unique behavior was demonstrated. Actinides are characterized by the progressive filling of the 5f electronic shell, which is the basis of their rich chemistry and physics.^[^ ^14^ ^]^ At their discovery in the ‘40s, the 5f shell was expected to show a similar behavior to the 4f shell, and therefore actinides were assimilated to lanthanides. However, the larger radial extent of the 5f shell over the 4f shell makes the former more prone to engage in bonding and more flexible in terms of accommodating electrons.^[^ ^15^ ^]^ This difference is readily seen in the set of stable oxidation states of lanthanides and actinides. Lanthanides are mostly found in the trivalent state, with only rare exceptions. In contrast, the first half of the actinide series exhibits a broader spectrum of oxidation states, reducing for the second half of the series, as shown in **Table** **1**. In this regard, actinides represent a unique set of chemical elements whose behavior when nanosized may differ from what is observed on more stable materials.

**Table 1:** Year of discovery, electronic configuration, and oxidation states of the actinide series. In bold, the most stable oxidation state.

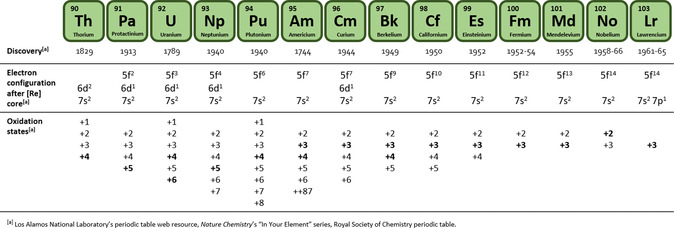

The interest in actinide‐bearing nanomaterials has grown considerably in the last decades, driven by the discovery that radionuclides can migrate over long distances in the form of colloids.^[^ ^16^ ^]^ Understanding and controlling the behavior of actinide systems at the nanoscale then became a matter of nuclear and environmental safety.^[^ ^17^ ^]^ Actinide‐bearing colloids can be pseudocolloids, formed by the sorption of actinide ions on colloidal mineral phases,^[^ ^16^, ^18^, ^19^, ^20^ ^]^ or intrinsic colloids, resulting from the strong tendency of tetravalent actinides to hydrolyze and form polynuclear species that aggregate to form colloids and NPs.^[^ ^21^ ^]^ These entities are part of the thermodynamic equilibrium and must be considered to correctly estimate the solubility and the mobility of radionuclides in the environment.^[^ ^22^ ^]^ Initially, actinide NPs were not yet a research topic in their own right. Rather, they were found in studies of actinide solubility and dissolution. Intrinsic colloids formed within these studies were identified as oxo‐hydroxide solid phases with poor crystallinity and ill‐defined structures. These conclusions were largely based on the absence of well‐defined X‐ray diffraction (XRD) patterns and on the poor structural order as determined from extended X‐ray absorption fine structure (EXAFS) measurements at the actinide site.^[^ ^21^, ^23^, ^24^ ^]^ At this early stage of the field, the work by Soderholm and coworkers on actinide polynuclear complexes was of fundamental importance to advance the understanding of the structure of actinide colloids and NPs. In particular, they synthesized and solved the structure of the [Pu_38_O_56_Cl_54_(H_2_O_8_)]^−14^ cluster, revealing the close similarity of its core to a 1.2 nm PuO_2_ NP.^[^ ^21^, ^25^ ^]^ The emerging evidence of the importance of actinide NPs in environmentally relevant scenarios made them become the primary object of a branch of actinide research, stimulating important progress on actinide NPs intentional synthesis.

The book “Actinide Nanoparticles Research,” edited by Kalmykov and Denecke, traces the state of the field around 2011.^[^ ^26^ ^]^ It reviews the characterization techniques, the state of theoretical modeling, and the environmental behavior of actinide NPs. In 2013, a thematic issue of Chemical Reviews dedicated to nuclear chemistry included four contributions relevant to the emerging field. The contribution of Altmaier and collaborators on aqueous actinide chemistry and thermodynamics,^[^ ^22^ ^]^ the review by Knope and Soderholm on the formation of polynuclear complexes and NPs as a product of actinide hydrolysis,^[^ ^21^ ^]^ the review by Walther and Denecke focused on actinide colloids and particles of environmental concern,^[^ ^17^ ^]^ and the contribution by Qiu and Burns on synthetic actinide clusters with oxide, peroxide, and hydroxide bridges.^[^ ^27^ ^]^ These excellent review articles show how actinide nanosystems emerged from various branches of actinide science.

These early works promoted interest in synthetic actinide NPs and in their structural characterization. Several groups started to investigate actinide NPs’ formation and their structural properties in correlation with reaction conditions.^[^ ^28^, ^29^, ^30^, ^31^, ^32^, ^33^, ^34^, ^35^, ^36^, ^37^, ^38^, ^39^, ^40^, ^41^, ^42^ ^]^ In parallel, efforts toward the accurate structural characterization through the use of synchrotron techniques and the development of analysis methods specific to NPs multiplied.^[^ ^43^, ^44^, ^45^, ^46^, ^47^, ^48^, ^49^ ^]^ The research efforts were directed to the understanding of their formation process, structural properties, and reactivity. Among the key questions that needed an answer, we can list: i) what is the oxidation state of actinide ions during the formation process and in the end product; ii) what are the growth paths and aging processes, and how are they affected by chemical parameters like pH, ionic strength, and concentration of complexants; and iii) how the sizes and the reactivity of the NPs depend on the synthesis method.

The rich portfolio of techniques available at synchrotron radiation sources offers exceptional tools to tackle these questions. Almost all synchrotron radiation sources allow measurements of actinides under specific safety regulations and within certain limits. Indeed, synchrotron techniques have been utilized to investigate actinide NPs since the beginning and have strongly contributed to advances in the field. The most commonly employed techniques are synchrotron XRD, applied to both single crystals and powders, and EXAFS, owing to their methodological maturity. Powder XRD (PXRD) is routinely used to estimate NP sizes. It is sensitive to the crystalline domains but blind to minor amorphous phases. EXAFS probes the local structure of the metal ions, providing accurate first and second shell distances and average coordination numbers.^[^ ^50^ ^]^ Its sensitivity is limited to the short‐range order, and strong structural disorder affects the estimated coordination numbers. Additionally, the pair‐distribution function (PDF) extracted from total X‐ray scattering is a very powerful technique for the structural characterization of NPs.^[^ ^51^ ^]^ The interatomic distances up to several tens of angstroms are extracted from the signal, making this technique sensitive to the short‐, medium‐, and long‐range order. PDF was extensively used by Soderholm and coworkers to gain insight into the atomic arrangement of actinide polynuclear complexes.^[^ ^52^ ^]^ Although a few recent studies have applied PDF analysis from high‐energy X‐ray scattering (HEXS) to actinide NPs,^[^ ^44^, ^46^ ^]^ the potential of this synchrotron‐based technique continues to be predominantly underexploited in the field. In recent studies, the use of small‐angle X‐ray scattering (SAXS) has increased.^[^ ^53^, ^54^, ^55^, ^56^ ^]^ This technique provides an estimation of the external size of the NPs and of their agglomerates. Another technique of growing importance for actinide science is high‐energy‐resolution fluorescence‐detected (HERFD) X‐ray absorption near edge structure (XANES).^[^ ^57^ ^]^ Since the exceptional sensitivity to actinide oxidation state was demonstrated,^[^ ^58^ ^]^ its use has constantly increased. In view of the results that will be presented later in the text, the following section is dedicated to HERFD XANES to illustrate its specificity to the actinide field and its use on actinide NPs.

## 2 HERFD XANES for Actinide NPs

HERFD XANES is a core‐level spectroscopy and, as such, is element selective and chemically sensitive. When applied to actinides, the most suitable edges to explore are the L_3_ and the M_4,5_, corresponding to a 2p_3/2_ excitation into unoccupied d‐states and to a 3d_3/2_ (M_4_) or 3d_5/2_ (M_5_) excitation into unoccupied f‐states. The L_3_ edge of actinides is in the hard X‐ray regime (16 300 eV for Th to 19 907 eV for Cf), while the M_4,5_ edges are in the tender X‐ray range (3332 eV for Th M_5_ to 4484 eV for Cf M_4_). The K edges are at very high energies, rarely reached at most beamlines, while N_4,5_ and O_4,5_ edges, which also probe the f‐states from the 4d and 5d, respectively, are in the soft and far‐UV range, requiring ultrahigh vacuum. Safety regulations impose sample containment, making measurements of actinides at low energies very challenging. The L_3_ and M_4,5_ edges are compatible with sample confinement. However, when measured with conventional XANES, the large lifetime broadening of the core‐hole (8.2 eV at U L_3_ and 3.5 eV at U M_4,5_ edges)^[^ ^59^ ^]^ smooths spectral features to an extent that renders the M_4,5_ edges almost featureless and poorly informative. The L_3_ edge remains informative, with the edge position shifting with the oxidation state and the extended postedge region used for EXAFS analysis.

**Figure 1:**
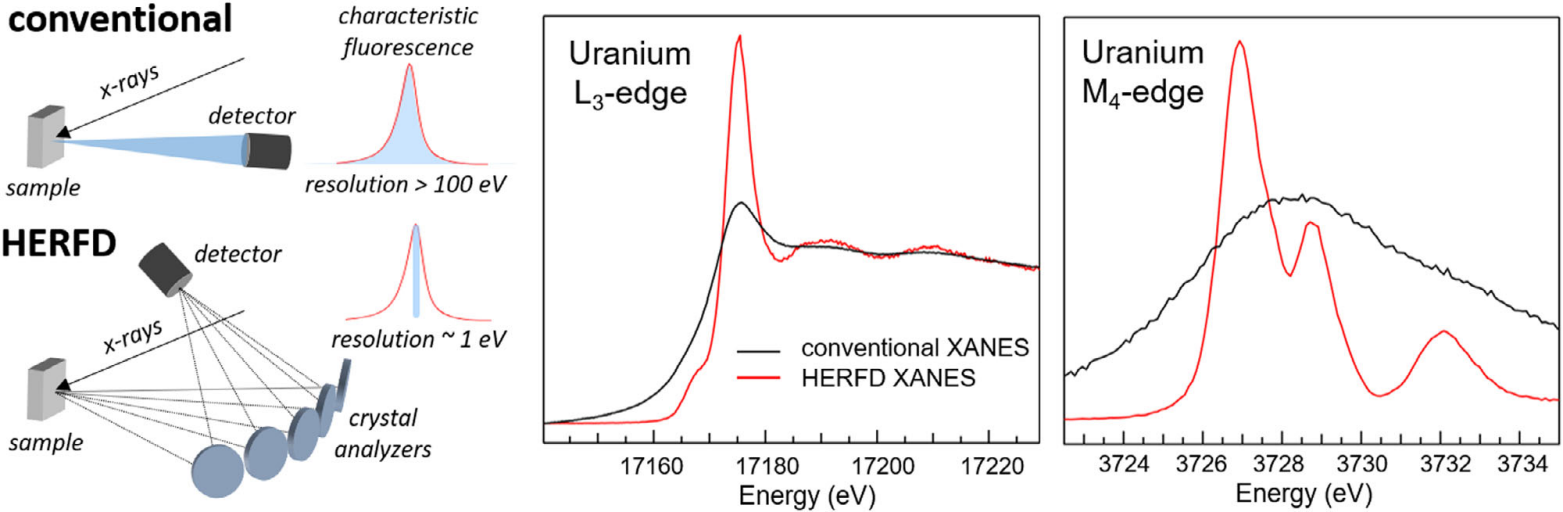
Scheme of the detection of conventional and HERFD XANES (left). Comparison of conventional and HERFD XANES of uranyl (UO_2_(NO_3_)_2_*6(H_2_O)) at the U L_3_ (middle) and M_4_ (right) edges.

With the advent of the HERFD detection mode,^[^ ^60^ ^]^ the limitations due to the large core‐hole lifetime broadening have been partly overcome. In this technique, an X‐ray emission spectrometer is used to analyze the characteristic fluorescence emitted by the sample with a resolution of the order of the eV. By integrating only the maximum of the characteristic fluorescence with a bandwidth smaller than the core‐hole lifetime broadening, spectral features are sharpened.^[^ ^61^ ^]^ As a comparison, conventional XANES acquired in fluorescence mode is obtained by integrating the full characteristic emission line with solid‐state detectors having ≈130 eV resolution. The core‐hole lifetime broadenings at the L_3_ and M_4,5_ edges of actinides are ≈7–9 eV and 3–4 eV, respectively. It follows that HERFD XANES spectra acquired with an X‐ray emission spectrometer of ≈1 eV resolution result in a significant improvement. **Figure** **1** schematically shows the two detection modes and the comparison of conventional and HERFD XANES at the L_3_ and M_4_ edges of U. Johann‐type X‐ray emission spectrometers are currently the solution of choice for HERFD on actinide systems.^[^ ^62^, ^63^, ^64^ ^]^ This type of spectrometer mounts several bent crystal analyzers to collect the emitted fluorescence over a large solid angle. It is well suited for actinide research, where the amount of radioactive element has to be kept as small as possible and within confinements. The energies of the M_β_ emission lines from Th to Pu, used to collect HERFD XANES at the M_4_ edge, are covered by two sets of crystal analyzers, that is, Ge(220) and Si(220), operated at Bragg angles between 80° and 65°. The resolution of a Johann‐type X‐ray emission spectrometer at the An M_β_ emission lines mounting crystal analyzers bent at a 1 m radius is ≈1 eV. This resolution is substantially smaller than the core‐hole lifetime broadening of the 3d_3/2_ hole of An, resulting in considerable sharpening of the HERFD XANES spectral features at the An M_4_ edge compared to conventional XANES. Figure 1 shows a sketch of a five‐crystal Johann‐type X‐ray emission spectrometer.

**Figure 2:**
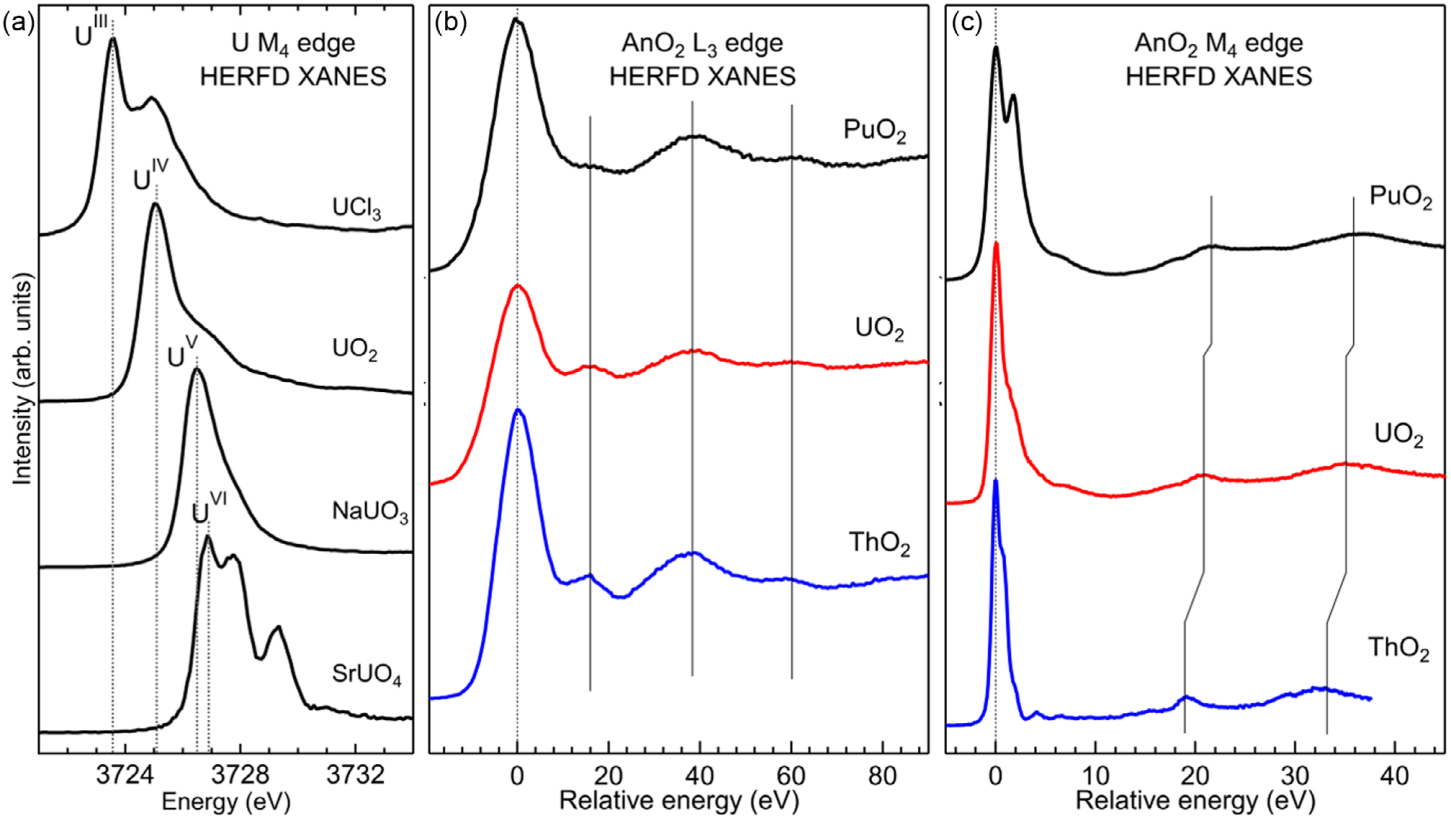
a) U M_4_ edge HERFD XANES of compounds with U in different oxidation states, reflected in the shift of the main absorption edge. b) L_3_ and c) M_4_ edge HERFD XANES of ThO_2_, UO_2_, and PuO_2_. The spectra are shifted to the maximum of the absorption to highlight the similarities of the postedge region, the signature of the same local structure.

For actinide M_4,5_ edges, the use of the HERFD detection mode has been demonstrated to be a game changer because of the extreme sensitivity of the absorption edge position to the oxidation states of actinides.^[^ ^57^, ^58^, ^65^ ^]^ The improved resolution enhances the sensitivity to changes of the oxidation state and of the local structure. Changes in the oxidation state are usually observed as shifts of the absorption edge, while the shape of the spectrum, especially in the postedge region, is a fingerprint of the local structure. **Figure** **2** illustrates these properties of the XANES spectra. Figure 2a shows the shift of the U M_4_ HERFD for different oxidation states of uranium. Figure 2b,c illustrate the L_3_ and M_4_ HERFD XANES of AnO_2_ (An = Th, U, Pu), which present a characteristic pattern in the postedge region. Actinide dioxides (AnO_2_) crystallize in the fluorite structure, where the actinide ion is surrounded by eight oxygen atoms arranged at the corners of a cube. The local structure around Th, U, and Pu is therefore the same, with distances that are proper to each material. This similarity is nicely reflected in the shape of the postedge regions of both L_3_ and M_4_ edges, where the characteristic pattern is the signature of the fluorite structure. With HERFD, all spectral features are better resolved, and the sensitivity to spectral changes is therefore enhanced.

The comparison of the L_3_ and M_4_ edge HERFD XANES of AnO_2_ in Figure 2b,c opens a fundamental question that gets to the heart of core‐level spectroscopy and AnO_2_ electronic structure: why is there a noticeable difference in the energy separation between the main edge and the characteristic postedge features in the actinide M_4_ edge (3d_3/2_ → 5f) spectra, while such a difference is absent at the L_3_ edge (2p_3/2_ → 6d)? There are many factors that can contribute to this difference. A key factor is the difference in the nature and localization of the probed states: 5f states are more localized, whereas 6d states are delocalized. As the system becomes less ionic and more covalent (with metal–ligand hybridization), the M_4_ edge may be more sensitive, and spectral features might shift accordingly, while similar changes would be less impactful on the L_3_ edge. The ThO_2_ with a 5f empty shell might show different hybridization behavior, reflected in M_4_ data, compared to UO_2_ and PuO_2_. Another key factor is the core‐hole effect, which differs between the two edges. In particular, the core‐hole at the L_3_ edge is screened by the 5f electrons. The energy position with respect to the Fermi energy of the 5f and 6d along the series may also affect this separation.

When applied to actinide NPs, HERFD XANES is used to gain insight into the oxidation state and the local structure, which are crucial factors to understand NPs stability, reactivity, and mechanism of formation. It is relevant to highlight that HERFD XANES, and generally all X‐ray absorption spectroscopies, are sensitive to all the atoms of the selected species within the probed volume. Additionally, each atom contributes equally to the signal, independently from the degree of crystallinity of its environment. Another advantage of hard X‐ray spectroscopies is their versatility: they can be applied to both solids and liquids, do not require ultrahigh vacuum, and are nondestructive. These characteristics are particularly relevant when applied to NPs because they are complementary to other techniques that are commonly used in the field. High‐resolution transmission electron microscopy (HRTEM) is used to visualize NPs when possible. Compared to HERFD XANES, HRTEM probes only a very limited portion of the sample, the electron beam is a considerably harsher probe than X‐rays, and ultrahigh vacuum coupled with the electron probe may cause sample alteration. PXRD is also among the commonly used techniques to characterize NPs. Differently from HERFD XANES, PXRD is sensitive to the coherent crystalline domains. Larger domains dominate the signal, making PXRD inadequate for determining the presence of amorphous phases and smaller particles. The PDF obtained from HEXS is among the most used techniques to characterize NPs, as previously mentioned. Differently from PXRD, by considering the total scattering instead of only the coherent scattering, all atomic pair distances are contributing to the signal. Therefore, interatomic distances from smaller crystalline domains as well as more disordered phases are probed. This makes the information extracted from PDF complementary to that obtained from EXAFS, which probes the short‐range order with a resolution of the order of a few tens of Å. While EXAFS focuses on the local environment, PDF probes both the short‐ and long‐range order. The complementarity of the techniques used to characterize NPs is fundamental to correlating results and building a consistent structural overview. In the following, we will review the progress in the domain of actinide NPs of PuO_2_, UO_2_, and ThO_2_. We will focus on progress obtained using HERFD XANES in correlation with other synchrotron techniques.

## 3 Actinide Dioxide NPs

### 3.1 Plutonium Dioxide NPs

Historically, PuO_2_ NPs became a matter of concern when Pu‐bearing colloids were identified as a highly mobile form of radionuclides in the environment. The first work on Pu colloids by Jacobson and collaborators dates back to 1948, a study on the uptake of Pu by soil.^[^ ^66^ ^]^ At that time, the authors were not aware that Pu was retained in the form of colloids. Almost 50 years later, Penrose^[^ ^4^ ^]^ and coworkers identified colloidally bound Pu and Am in monitoring wells at 3.4 km downstream from the site where contaminated liquid waste was released, and Kersting and coworkers reported the transport of Pu associated with colloids at the Nevada Test Site in 1991.^[^ ^6^ ^]^ These were the first works raising the alarm about actinide colloids as vectors of radionuclide contamination in the environment. In 1992, Bates^[^ ^67^ ^]^ and coworkers reported the formation of insoluble Pu‐ and Am‐bearing colloidal particles during simulated weathering of a high‐level nuclear waste glass. These findings raised the alarm that models of actinide mobility and repository integrity, which assume complete solubility of actinides in groundwater, underestimate the potential for radionuclide release into the environment and suggested that colloid formation should be considered to meet long‐term performance specifications. Pu‐bearing NPs can be released or formed in the environment as side products of various processes, including nuclear accidents, weapons testing, and long‐term waste disposal. Understanding their formation mechanism, behavior, mobility, and transformation is therefore critical for assessing long‐term environmental risks and developing remediation strategies. More recently, the possibility of nanosizing PuO_2_ to improve thermal conductivity, radiation resistance, and fission product retention is being investigated for fast breeder reactors and some research reactors.

Modeling Pu chemical evolution and migration in the form of colloids/small NPs is particularly challenging. Pu is stable in oxidation states from III to VI in environmentally relevant conditions, and multiple oxidation states can coexist. As with all tetravalent actinides, Pu(IV) strongly hydrolyzes in aqueous solutions,^[^ ^21^, ^22^ ^]^ resulting in the formation of polynuclear species and intrinsic colloids that may form a stable suspension or precipitate. This complex chemical behavior, combined with the variety of redox conditions met in the environment (pH, mineral interfaces, bacteria, etc.), makes Pu geochemistry particularly complex.^[^ ^68^ ^]^ With a series of works published in the early 2000s, the Nuclear Institute in Karlsruhe advanced considerably the understanding of plutonium aqueous chemistry through comprehensive experimental investigations on plutonium solubility and hydrolysis, confirming the relevance of colloid formation.^[^ ^69^, ^70^, ^71^, ^72^, ^73^, ^74^ ^]^

The environmental risk represented by PuO_2_ NPs has prompted numerous research groups into new studies. Different synthesis routes have been explored, and structural characterization has been conducted with a multitechnique approach.^[^ ^28^, ^34^, ^36^, ^39^, ^44^, ^75^, ^76^ ^]^ The recent advances in the synthesis and characterization of PuO_2_ NPs have been extensively reviewed by Virot and collaborators.^[^ ^77^ ^]^ The works on Pu oxide nanoclusters, initiated by Soderholm and collaborators, marked a fundamental step in the understanding of the structure of Pu NPs at the molecular level.^[^ ^25^, ^78^, ^79^, ^80^ ^]^ The structure of these well‐defined polynuclear clusters was determined by single‐crystals XRD, providing an atomic description of polynuclear species that are expected to be very similar to those formed during Pu(IV) hydrolysis. Based on the structure of the Pu{38} oxide nanocluster, [Pu_38_O_56_Cl_54_(H_2_O_8_]^−14^, which has a PuO_2_ core, Soderholm and collaborators challenged the accepted idea that the products of Pu hydrolysis are ill‐defined hydrous oxide phases, but rather nanometric‐sized particles of PuO_2_.^[^ ^25^ ^]^ The recent work by Cot‐Auriol and collaborators, who found the signature of a Pu(IV) oxo‐hydroxo hexanuclear cluster during the formation of 2 nm size PuO_2_ NPs, confirms the relevance of polynuclear clusters as intermediates to colloid formation.^[^ ^80^ ^]^

The use of X‐ray synchrotron techniques has a prominent role in the structural characterization of PuO_2_ NPs. Pu L_3_ EXAFS has been used to determine Pu—O and Pu—Pu distances and to deduce the presence of Pu oxidation states other than Pu(IV). Conradson and collaborators found three different Pu—O distances and deduced the presence of Pu(V) in the NPs.^[^ ^81^ ^]^ Other authors also found the broad distribution of the Pu—O bonds but ascribed it to structural disorder correlating with decreasing size.^[^ ^69^, ^75^ ^]^ Gerber and collaborators obtained an excellent fit of Pu L_3_‐edge EXAFS on ≈2 nm NPs with a single Pu—O distance and reproduced the asymmetry of the Pu—O shell with Monte Carlo simulations based on the PuO_2_ structure.^[^ ^44^ ^]^

A more direct probe of the oxidation state is provided by Pu M_4,5_ HERFD XANES, as demonstrated in the works by Gerber and collaborators.^[^ ^44^, ^82^ ^]^ The authors synthesized NPs from Pu(III), Pu(IV), Pu(V), and Pu(VI) precursors by chemical precipitation at close to neutral and alkaline pH (8 and >10). Interestingly, they found that all reaction paths lead to PuO_2_ NPs of 1.5–2.5 nm average size. The reaction path and formation time were found to be very similar for all precursors, except for Pu(VI). The latter showed an exceptionally slow reaction, proceeding through the formation of an intermediate phase after one week before reaching the final product after 6 weeks of reaction.^[^ ^28^ ^]^ Based on HERFD XANES at the L_3_ and M_4_ edges, the final precipitate was identified as PuO_2_ NPs, while the intermediate phase was identified as a pentavalent amorphous phase.^[^ ^83^ ^]^ In their works, the authors used a set of synchrotron techniques to assess the size, structure, and oxidation state of the NPs.

**Figure 3:**
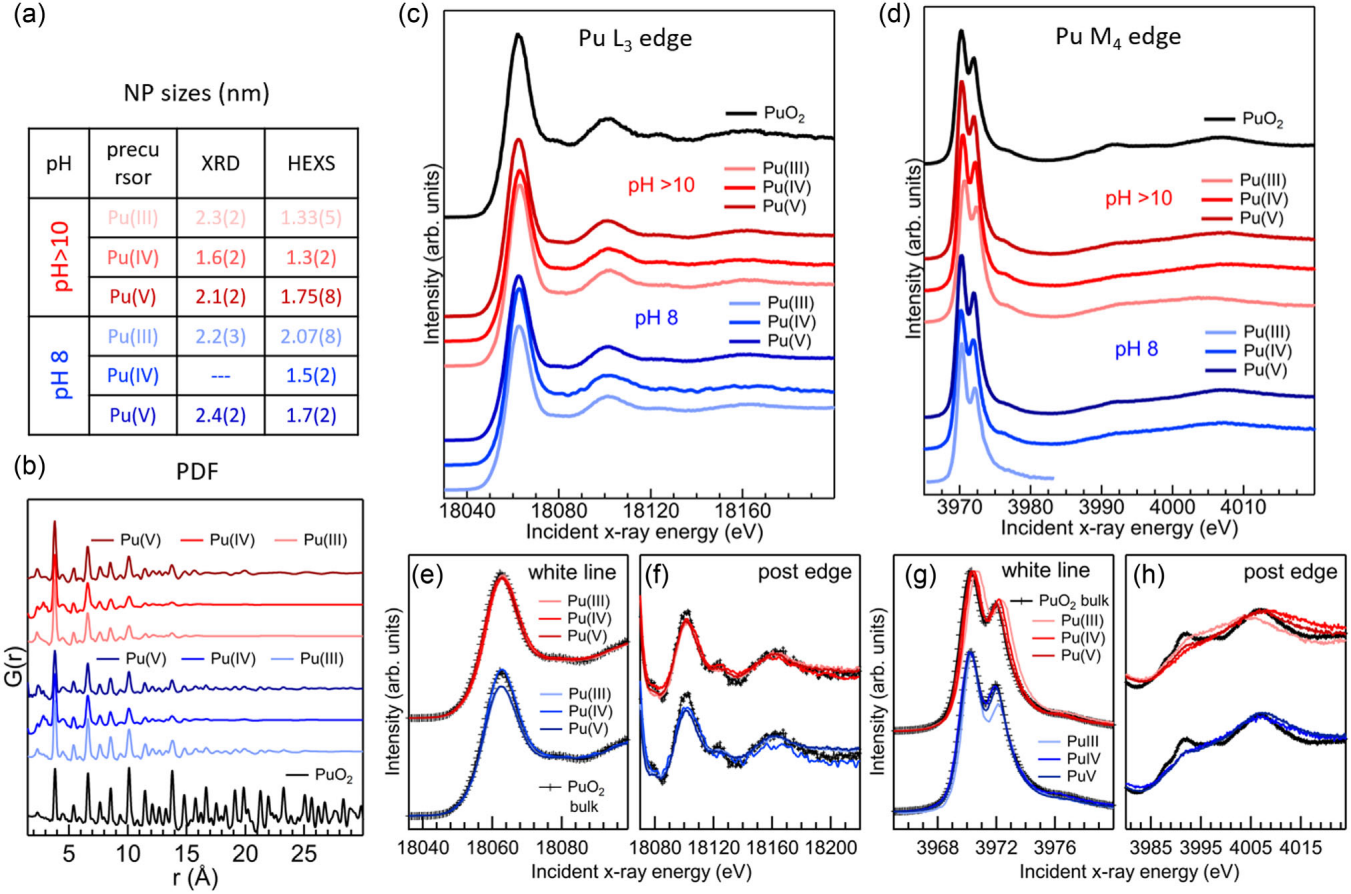
a) Table with PuO_2_ NPs average sizes from PXRD and HEXS and b) plot of the G(r) from HEXS. c) HERFD XANES at the L_3_ d) and at the M_4_ edge recorded on PuO_2_ NPs from different Pu precursors and compared to the spectra of bulk PuO_2_. e–h) Magnifications of the edge and postedge regions for both edges are shown with superimposed spectra. Adapted with permission.^[^ ^44^ ^]^ Copyright 2020, Royal Society of Chemistry.

The average sizes and the phase were estimated with PXRD, HRTEM, and PDF obtained from HEXS. **Figure** **3**a reports the table with the results from XRD and PDF and the plot of the G(r) from HEXS data. All techniques determined that the precipitate consisted of PuO_2_ crystalline NPs of average diameters below 3 nm, with HRTEM and PXRD results systematically larger than PDF. The differences are understood considering the different sensitivity of the techniques to long‐ and medium‐range order. PXRD mostly probes long‐range order, and PDF is sensitive to short‐, medium‐, and long‐range orders.

The oxidation state of Pu and the potential presence of secondary amorphous phases were evaluated with HERFD XANES at the L_3_ and M_4_ edges. In the case of Pu(III), Pu(IV), and Pu(V) precursors, the Pu M_4_ edge spectra were compatible with pure Pu(IV), excluding the presence of significant amounts of other oxidation states. Additionally, the shape of both Pu M_4_ and L_3_ HERFD XANES unequivocally determined that the only phase present was PuO_2_. Figure 3c,d reproduces the Pu L_3_ and M_4_ HERFD XANES results from Gerber and coauthors.^[^ ^44^ ^]^ The NPs spectra closely resemble that of the PuO_2_ reference. For both edges, the position of the absorption edge and the spectral features in the postedge region are the same for bulk PuO_2_ and the NPs obtained with different synthesis parameters. This is a strong indication that the nature of the final products is PuO_2_. To better illustrate this correspondence, Figure 3e–h shows magnification of the edge and postedge regions with superimposed spectra. Inspection of the postedge region reveals that NPs spectral features are broader compared to the PuO_2_ bulk reference, especially at the M_4_ edge. This effect is often observed on XANES of NPs and is contributed to by static disorder and by surface and subsurface atoms having truncated local structure compared to atoms in the bulk. This effect is therefore compatible with NPs below 2 nm, for which the contributions of surface and subsurface atoms become dominant. A similar effect was also observed on Pu M_5_ HERFD XANES on Pu colloids.^[^ ^84^ ^]^

The same authors also investigated the aging of the PuO_2_ NPs obtained through chemical precipitation. After 3 months, the PuO_2_ NPs were remeasured and were found unchanged, with no detectable size variations and changes in the oxidation state, differently from UO_2_ and ThO_2_ NPs that are discussed later in the text. Gerber and collaborators expanded their investigation to include the formation of PuO_2_ NPs in acidic pH. As for the previously investigated pH values, all reactions led to the formation of ≈2 nm PuO_2_ NPs.^[^ ^82^ ^]^ In this study, the authors highlighted the importance of considering traces of the mother solution when performing X‐ray spectroscopy. PuO_2_ NPs synthesized at pH 1 form a colloidal suspension and do not precipitate. The starting solution was therefore present during HERFD XANES characterization, resulting in the detection of 10% Pu(III), which could be erroneously attributed to Pu(III) in the structure. By monitoring the UV–vis signal during synthesis, the authors demonstrated that the residual signal of Pu(III) did not come from Pu atoms incorporated in the NPs.

### 3.2 Uranium Dioxide NPs

Early investigations of uranium colloids and NPs focused on the formation of uranium‐bearing colloids in aqueous environments,^[^ ^85^, ^86^, ^87^, ^88^, ^89^, ^90^ ^]^ particularly under conditions relevant to uranium mining and nuclear waste repositories.^[^ ^91^, ^92^, ^93^, ^94^, ^95^, ^96^, ^97^, ^98^ ^]^ As for plutonium, the formation of uranium‐bearing colloids alters the scenario of radionuclide transport by introducing a highly mobile form of U(IV) in the environment. For uranium, before the concern for colloids was raised, U(VI) was considered as the major risk for uranium transport in the environment due to its high solubility. Remediation strategies were then focused on U(VI) immobilization through its reduction into insoluble U(IV) phases. The microbial reduction of U(VI) into U(IV) insoluble phases has long been considered a potential path for U(VI) immobilization. However, several studies found that the end product of microbial reduction of U(VI) is UO_2_ NPs.^[^ ^91^, ^95^, ^96^ ^]^ Similarly, U‐bearing NPs can also form through the interaction with mineral phases. Over time, research expanded toward detailed local and electronic structure characterization of uranium NPs, using spectroscopic, scattering, and microscopy techniques.^[^ ^19^, ^99^, ^100^, ^101^ ^]^

The group of Bernier‐Latmani recently reported the observation of UO_2_ NPs formation from U(VI) reduction by magnetite.^[^ ^19^ ^]^ By employing spectroscopic and microscopy methods, the authors observed different steps of the reaction. Microscopy techniques revealed the fast adsorption of U(VI) onto magnetite NPs, followed by the formation of UO_2_ NPs that self‐aggregate into nanowires, which in turn collapse into nanocrystalline UO_2_ once a critical mass is reached. The insight obtained with U M_4_ HERFD XANES revealed that NP formation is accompanied by a slow process of U(VI) reduction to U(V) and subsequently to U(IV). The pentavalent state of U is elusive to most techniques. The U M_4_ HERFD XANES is very effective in determining its presence, and its use is playing a pivotal role in showing the importance of U(V) as an intermediate step in uranium chemistry.

Neil and collaborators provided a thorough characterization of U(IV)‐silicate colloids synthesized under conditions relevant to spent nuclear fuel storage and radioactive waste disposal.^[^ ^53^ ^]^ The authors determined the size, stability, and structure of the NPs using a large set of structural techniques. Their results indicate that U(IV)‐silicate colloids consisted of NPs of >10 nm made by a small crystalline UO_2_ core and a poorly ordered U(IV)‐silicate shell. The U(IV)‐silicate shell stabilizes the NPs in the high pH range investigated, providing a pathway to U mobilization. The complementarity of the structural techniques used in their work was crucial to reach a global and comprehensive description of the NPs structure, providing an excellent example of how to tackle a complex structural characterization.

Elucidating the fundamental properties of uranium colloids, including their size‐dependent electronic structure, surface reactivity, and aggregation dynamics, is critical for modeling uranium behavior in complex environments such as nuclear waste repositories, contaminated sites, and nuclear reactor systems.^[^ ^90^ ^]^ In these regards, the ability to control NPs properties through synthesis is a necessary prerequisite for further understanding. The work by Hudry and collaborators explored the nonaqueous synthesis of NPs of UO_2_, ThO_2_, and their mixed oxides.^[^ ^37^, ^38^, ^39^, ^40^ ^]^ They obtained well‐defined monodispersed, single‐domain NPs of different shapes and explored the effects of the precursor on the final products. In a recent work, the effects of precursor identity and surfactant concentration on the shape of UO_2_ NPs were investigated, and the authors achieved the synthesis of UO_2_ nanocubes of 4 nm under specific chemical parameters.^[^ ^102^ ^]^ Burns and coworkers developed the aqueous synthesis of uranyl‐containing cage clusters,^[^ ^103^, ^104^, ^105^, ^106^ ^]^ and the synthesis of several uranium molecular clusters extending to the nanoscale has been reported.^[^ ^107^, ^108^, ^109^, ^110^, ^111^, ^112^ ^]^ Cot‐Auriol and collaborators reported the synthesis of hexavalent uranium colloids by sonochemical methods with a schoepite‐like structure,^[^ ^113^ ^]^ while Moreau et al. reported the structural characterization of ultrasmall UO_2_ and ThO_2_ NPs obtained by employing a covalent organic framework as an inert template.^[^ ^42^ ^]^

Differently from PuO_2_, UO_2_ is prone to oxidation, and, as previously mentioned, the solubility of uranium depends on its oxidation state. It is therefore essential to understand the redox processes at play in the formation of UO_2_ NPs to determine their stability and reactivity. Underground nuclear waste repositories correspond to reducing and anoxic conditions, but the possibility of water infiltration would bring oxygen into the scenario. It seems, therefore, reasonable to pursue the understanding of UO_2_ NPs formation and evolution under a large set of chemical conditions.

**Figure 4:**
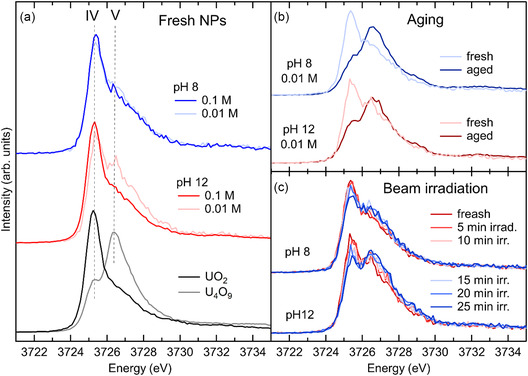
a) U M_4_ edge HERFD XANES of UO_2_ NPs synthesized at pH 8 and 12 compared to UO_2_ and U_4_O_9_. b) effect of aging the NPs and c) of irradiating the NPs with the X‐ray beam. Adapted with permission.^[^ ^29^ ^]^ Copyright 2021, Chinese Chemical Society (CCS), Peking University (PKU), and the Royal Society of Chemistry.

Gerber and collaborators conducted a thorough investigation on UO_2_ NPs formation and stability.^[^ ^29^ ^]^ With a similar approach used in their study of PuO_2_, the authors synthesized UO_2_ NPs of a diameter smaller than 3 nm from U(IV) aqueous solution and in reducing conditions. Independent from the initial reaction conditions, the as‐synthesized precipitates were UO_2_ NPs of size between 1.7 and 2.5 nm, as determined from PXRD and HRTEM. These techniques cannot determine the presence of other uranium binary oxides that could be present as amorphous phases in minor quantities. Authors measured U M_4_ edge HERFD XANES to determine the oxidation state of uranium and the possible presence of secondary phases in freshly precipitated NPs. **Figure** **4** is a summary of their HERFD XANES results. Figure 4a shows U M_4_ edge HERFD XANES on fresh UO_2_ NPs in comparison with the spectra of bulk UO_2_ and U_4_O_9_ as references for pure U(IV) and of U(IV)/U(V) mixture, respectively. At the U M_4_ edge, these oxidation states have the maximum of absorption at increasing energy going from U(IV) to U(V) (see Figure 2), and the presence of multiple oxidation states is easily detected by HERFD XANES. The HERFD XANES spectra of UO_2_ NPs obtained at different pH and concentrations closely resemble the spectrum of bulk UO_2_, characterized by a sharp and intense peak at the absorption edge and by a shoulder on the high‐energy side of the main peak. However, in UO_2_ NPs, the high‐energy shoulder is more pronounced, possibly hinting at the presence of a small amount of U in higher oxidation states. Indeed, theoretical calculations indicate that the growth of the shoulder is also compatible with the presence of U(IV) with different local symmetry and coordination from that of U(IV) in UO_2_.^[^ ^114^ ^]^ EXAFS measurements by the authors on the same UO_2_ NPs excluded the presence of U—O bonds with U(V) and U(VI) and indicated the presence of strong disorder and an average coordination number for the first O shell reduced compared to bulk UO_2_. EXAFS results therefore support the interpretation of the increased intensity of the shoulder as due to U(IV) atoms with local coordination different from U in bulk UO_2_, presumably at the surface of NPs.

The same authors investigated the stability of UO_2_ NPs. The collection of U M_4_ HERFD XANES on fresh samples already gave an important indication about their high reactivity. Under prolonged X‐ray irradiation in air, the intensity of the high‐energy shoulder of HERFD XANES corresponding to the U(V) oxidation state substantially increased with a simultaneous decrease of the peak of U(IV). Spectra acquired consecutively on the same spot are shown in Figure 4c. The progressive decrease of the U(IV) peak in favor of the growth of higher energy peaks is indicative of oxidation toward U_4_O_9_. The authors had to use a dedicated airtight sample holder and reduce the time of exposure to acquire spectra reflecting the structure of fresh NPs. Interestingly, the degradation observed under X‐ray exposure turned out to be similar to the effect of aging the NPs. Some samples were left for 2 years sealed in plastic tubes with some residual water after having been washed and were measured afterward to determine NPs stability. The size of aged NPs was estimated from PXRD and HRTEM to be increased to 6 nm. Notably, the similarity of the XRD patterns of different uranium binary oxides and the broadening of XRD peaks due to the reduced size make it impossible to distinguish and quantify different uranium oxide phases from XRD. The exceptional sensitivity of U M_4_ HERFD XANES to uranium's different oxidation states allows to clarify this point even on amorphous materials. The spectra of fresh and aged UO_2_ NPs are shown in Figure 4b. These data indicate that UO_2_ NPs tend to transform to U_4_O_9_ over time, probably through dissolution and recrystallization processes.

### 3.3 Thorium Dioxide NPs

The studies on ThO_2_ NPs are mostly related to the use of Th(IV) as a surrogate for heavier An(IV), which makes the behavior of ThO_2_ relevant for the general understanding of nanosized AnO_2_. As for PuO_2_ and UO_2_, the first NPs of concern have been Th(IV) intrinsic colloids, whose formation and relevance were determined in the framework of Th(IV) solubility studies. The presence of 1–2 nm colloids as part of the aqueous equilibrium species was determined in the early 2000s.^[^ ^5^, ^115^, ^116^, ^117^, ^118^, ^119^, ^120^ ^]^ The solid phase isolated by ultracentrifugation or precipitation was determined to be amorphous based on XRD. In parallel, the Th local structure as deduced from EXAFS analysis was found to be made by a disordered Th—O sphere, low Th—Th coordination numbers, and Th—O and Th—Th distances scattered around the values of crystalline ThO_2_. Based on these structural methods, Th(IV) intrinsic colloids have been identified as ill‐defined, poorly crystalline Th(IV) oxo‐hydroxide phases. The nomenclature for these ill‐defined solid phases is not uniform across the literature on the subject and has been named Th(OH)_4_(am), ThO_2_·xH_2_O(am), ThO_2_(am, hyd), and ThO_2_(am). In the framework of Th(IV) solubility studies, the aging of the fresh precipitates has also been extensively investigated.^[^ ^23^, ^24^, ^121^, ^122^ ^]^ The experiments span a large range of synthesis conditions and hydrothermal treatment. However, all results pointed to the tendency of the ill‐defined Th oxo‐hydroxo phases to transform into nanocrystalline ThO_2_. The recent work from Kiefer and collaborators provides a comprehensive overview of the work done on these Th(IV) precipitates and provides novel results on the structure, solubility, and thermodynamics of fresh and aged ThO_2_(am, hyd).^[^ ^24^ ^]^ The authors find that the pH and the time of the aging process do not affect the structure of the end products. In all cases the crystallinity of the fresh ThO_2_(am, hyd) increases and tends to that of bulk ThO_2_. Interestingly, despite no differences being found in the structure of the aged precipitates, as probed by a multimethod approach, the solubility results revealed an important impact of the aging time and pH. These apparently contradictory results are rationalized by the authors as evidence that Th(IV) solubility is controlled by a 0.2–0.4 nm surface layer, whose behavior is affected by the aging conditions but whose characterization remains elusive to the structural techniques employed. Similar conclusions were found in a recent work on CeO_2_ NPs dissolution.^[^ ^123^ ^]^ Plakhova and collaborators correlate the anomalous results on 2 nm NPs to the different hydroxylation of the surface. Interestingly, indications of the different hydroxylation of the surface of 2 nm NPs were found in the Ce L_3_ HERFD XANES spectra and in XRD.

The formation of intrinsic Th(IV) colloids was marginally discussed also in the review by Knope and Soderholm dedicated to hydration, hydrolysis, and condensation reactions of actinides in solution.^[^ ^21^ ^]^ In their review, the authors aim at the metrical description of actinide complexes and aggregates identified by thermodynamic studies at the molecular level. To achieve this molecular‐level description, their work is built on the authors’ expertise in stabilizing and crystallizing polynuclear complexes formed in aqueous and nonaqueous chemistry and on the use of a portfolio of X‐ray techniques that directly probe metal–ligand coordination numbers and distances, namely single‐crystal XRD, EXAFS, wide‐angle X‐ray scattering, and PDF from HEXS. This portfolio of techniques probes short‐, medium‐, and large‐range order, providing an atomic description of these aggregates at all relevant length scales. Studies on the ill‐defined Th(IV) precipitates and their aging, making use of the complementarity of these X‐ray techniques, have evidenced the presence of medium‐range order compatible with nanocrystalline ThO_2_.^[^ ^124^ ^]^ In this new light, the amorphous nature of the Th(IV) ill‐defined precipitate originates from the structural characterization probing only the short (EXAFS) and long (PXRD) range order, missing the medium‐range order.

**Figure 5:**
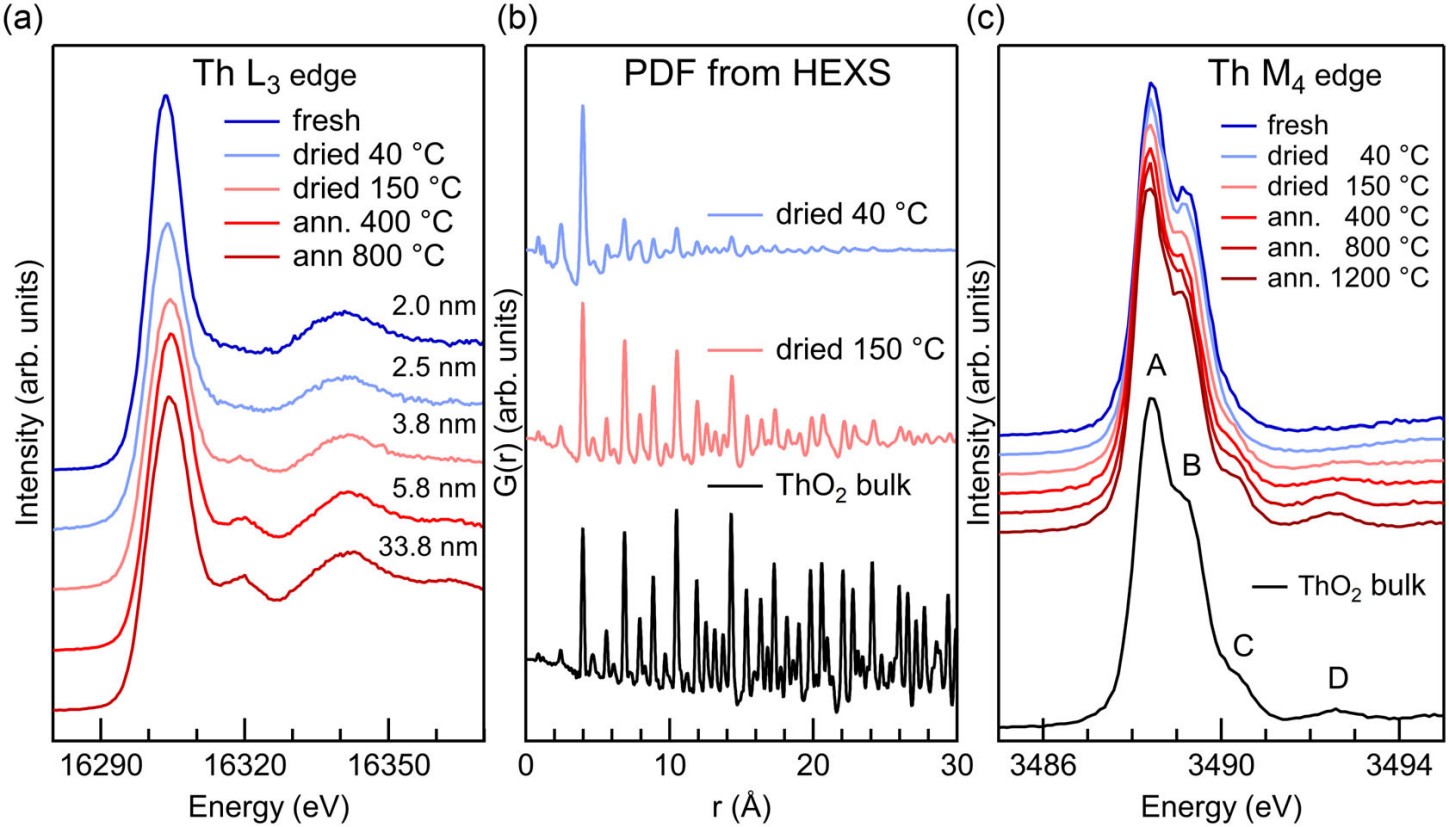
a) Th L_3_ edge HERFD XANES of ThO_2_ NPs of increasing size from top to bottom. b) PDF data of three selected samples. c) Th M_4_ edge HERFD XANES of ThO_2_ NPs of increasing size from top to bottom. Adapted with permission.^[^ ^45^, ^46^ ^]^ Copyright 2019, PCCP Owner Societies, and Copyright 2021, Wiley.

Starting from 2012, the studies dedicated to the intentional synthesis of ThO_2_ NPs multiplied, with the aim of understanding their aggregation behavior, stability, and surface chemistry.^[^ ^23^, ^34^, ^36^, ^38^, ^40^, ^42^, ^75^, ^125^, ^126^ ^]^ Moreau and coauthors were able to synthesize ultrasmall (<3 nm) ThO_2_ and UO_2_ NPs using the pores of a covalent organic framework as an inert template.^[^ ^42^ ^]^ The thorough structural characterization comprising transmission electron microscopy (TEM), SAXS, XANES, and EXAFS revealed that the fluorite structure is retained and particles are indeed nanocrystalline ThO_2_ and UO_2_. The ultrasmall size of the particles makes the contribution of surface atoms a relevant part of the experimental signal. The authors were therefore able to extrapolate structural information on the surface layers and found a broader distribution of Th—O and U—O distances centered on the average values of the corresponding fluorite structures. Plakhova and collaborators were able to synthesize nanocrystalline ThO_2_ NPs from 2.5 to 38 nm with chemical precipitation followed by hydrothermal treatments and annealing.^[^ ^126^ ^]^ Interestingly, the detailed characterization with XRD, HERFD XANES at Th L_3_ and M_4_ edges combined with the PDF from HEXS provided new insight into the formation process of ThO_2_ complexes and aggregates.^[^ ^46^ ^]^ The results from PDF and HERFD XANES are shown in **Figure** **5**. XRD analysis indicated that as‐precipitated products are amorphous phases whose crystallinity progressively increases with hydrothermal treatments and annealing. From HRTEM images, small NPs with ordered atomic planes were distinguished in the as‐precipitated phase. The measured interlayer distances and diffraction patterns were compatible with crystalline ThO_2_. In the spectra of Th L_3_ edge HERFD XANES, a postedge feature was missing for the smallest NPs, as shown in Figure 5a. Analysis based on calculated spectra from NPs structures revealed the pronounced sensitivity of this particular feature to Th—Th coordination, hinting at very small NPs.^[^ ^45^ ^]^ The PDF extracted from HEXS for the fresh precipitate mildly dried at 40 °C, as shown in Figure 5b, is characterized by a very intense peak at 3.96 Å. On the spectroscopy side, the Th M_4_ edge HERFD XANES of the fresh and mildly dried precipitate misses two features compared to bulk ThO_2_, that is, peak C and D (Figure 5c).

The analysis of the PDF data using ensembles of NPs of different sizes revealed that the 40 °C sample is mainly composed of Th hexamer‐like particles, very similar to Th hexamers reported in literature.^[^ ^127^, ^128^ ^]^ Calculations of the HERFD XANES spectra based on Th hexameric models confirmed that the absence of feature C is also explained by the presence of Th hexamers. More specifically, feature C is lost because of the broken cubic symmetry at the Th atoms constituting the hexameric unit. These studies added experimental evidence to the role of polynuclear complexes in the growth process of AnO_2_ NPs and confirmed the importance of using a multimethod approach to probe all the relevant length scales of the system.

Plakhova and collaborators also investigated the aging of the ThO_2_ X‐ray amorphous phase obtained by chemical precipitation.^[^ ^23^ ^]^ The isolated solid phase was aged at room temperature for 6, 18, and 36 months at different pH values, from acidic to alkaline. In their comprehensive study, they measured the solubility and characterized the structure of the solid phases. They used dynamic light scattering to measure the size of the aggregates and their stability. High‐resolution cryo‐TEM and synchrotron XRD were used to characterize the size and structure. Their results, in line with the existing literature, indicate that progressive increase in the crystallinity with the aging process. The authors found that the acidity of the solution plays a critical role in determining the growth process toward larger ThO_2_ nanocrystals. They proposed two different mechanisms for ThO_2_ crystallization. A mechanism of dissolution and recrystallization at pH < 5, favored by the higher solubility of Th(IV), and a process based on oriented attachment at higher pH. The same authors recently investigated the fate of ThO_2_ NPs in aqueous phosphate‐rich environments, finding the formation of a hydrated double sodium‐thorium phosphate in analogy with similar investigations on CeO_2_.^[^ ^129^, ^130^ ^]^ In a work dedicated to the comparative analysis of the O K‐edge XANES of CeO_2_ and ThO_2_, Amidani and collaborators explored theoretically the sensitivity of this edge to the nanosize, with an approach recalling their work at the Th L_3_‐edge, which shows the absence of the first postedge feature after the white line for ThO_2_ NPs compared to bulk ThO_2_.^[^ ^45^ ^]^ Their results suggest a strong sensitivity of the O K‐edge to changes at the surface.^[^ ^131^ ^]^

## 4 Comparing AnO_2_ NPs

The picture emerging from the studies reported so far on the chemical and physical properties of actinide NPs outlines similarities and differences. The synthesis of NPs by aqueous chemistry methods for the three actinide dioxides considered here, that is, ThO_2_, UO_2_, and PuO_2_, always results in NPs below ≈3 nm. Their structure, when thoroughly characterized at all relevant length scales, closely resembles that of their bulk AnO_2_, already for sizes below 2 nm. Aging processes increase the crystallinity and the size of the products, with a mechanism that can be influenced by chemical parameters. The three AnO_2_ differ, however, in their redox behavior. While for ThO_2_ and PuO_2_ only the An(IV) state is found and no changes are observed over time, UO_2_ NPs, while having a similar formation path, tend to oxidize toward U_4_O_9_, in line with the tendency of UO_2_ to accommodate excess O. This comparison is particularly relevant for UO_2_ and PuO_2_, since both cations have several stable oxidation states. It becomes relevant to compare these results with the CeO_2_ NPs, since Ce is stable in the III and IV oxidation states and, most importantly, is used as an analog for several An(IV) cations. Interestingly, similar aqueous synthesis of CeO_2_ NPs and detailed characterization of Ce polynuclear clusters found no evidence of Ce(III), pointing toward a behavior more similar to PuO_2_.^[^ ^132^, ^133^, ^134^ ^]^ Romanchuk and collaborators conducted a comparative work between PuO_2_ and CeO_2_ NPs and indeed concluded that CeO_2_ is an appropriate analog of PuO_2_.^[^ ^49^ ^]^ To complete the picture of AnO_2_ NPs synthesis, structure, and redox behavior, more investigations on NpO_2_ NPs are desirable. Currently, the only work on the intrinsic formation of NpO_2_ NPs confirmed the similar behavior to the AnO_2_ discussed in this work.^[^ ^135^, ^136^ ^]^ The relevance of polynuclear clusters during the initial steps of An(IV) hydrolysis and NPs formation is also a common point to all AnO_2_ investigated so far. Their presence in the initial stages of formation has been experimentally confirmed for ThO_2_ and PuO_2_ and is expected to be relevant also for UO_2_.

From the data available on other synthesis methods for AnO_2_ NPs, it is difficult to extract similar systematics. Hudry and collaborators reported the effects of the precursor type on the final products in nonaqueous surfactant‐assisted synthesis of UO_2_ and ThO_2_ NPs.^[^ ^38^ ^]^ UO_2_ and ThO_2_ NPs obtained by using a covalent organic framework as an inert template had very similar structural properties.^[^ ^42^ ^]^ The comparison between NPs obtained by sonochemical and hydrolytic methods revealed similar structures, characterized by a more disordered surface.^[^ ^32^, ^76^ ^]^

EXAFS analysis of small NPs has often been exploited to gain insight into the structure of the surface in view of indirectly determining the presence of atoms in different oxidation states at the surface. When EXAFS analysis is performed with advanced methodologies,^[^ ^44^ ^]^ sometimes specifically designed to extract surface information,^[^ ^47^, ^49^ ^]^ and complemented with other techniques, the results indicate that surface disorder is not correlated with the presence of oxidation states other than the tetravalent.^[^ ^77^ ^]^ This highlights the importance of developing analysis strategies and/or exploiting the different sensitivities of X‐ray techniques to extract a consistent and reliable picture of the NPs surface.^[^ ^53^, ^80^ ^]^

## 5 Conclusion and Outlooks

The interest in actinide NPs was initiated by the concern that they are a highly mobile form of actinide transport in the environment. It further developed toward more fundamental aspects, in particular the understanding of their formation processes, stability, and reactivity. More recently, nanostructured actinide materials have been looked at with interest for their potential use as future nuclear fuels with improved properties. Future progress in the field is expected from the development of new synthesis procedures and from the exploration of new systems, in particular NpO_2_, mixed oxides, and doped NPs.

The application of new methodologies and sophisticated analysis tools to deepen the understanding of the physicochemical properties of actinide NPs is also a potential direction to expand the field and advance the knowledge. Some of the works reviewed here designed original approaches to analyze EXAFS, PDF, and HERFD XANES data with promising results in terms of new structural insight.^[^ ^45^, ^46^, ^47^, ^49^ ^]^ Broadening the use of these analysis strategies to the investigation of actinide NPs is desirable in the near future. For further progress, the actinide field should look at what has been developed for nanoscience of nonradioactive elements, where the application of complex modeling and machine learning stands out as the most relevant recent advancement to get structural insight.^[^ ^137^, ^138^, ^139^ ^]^

In parallel with improved structural characterization capabilities, the research on actinide NPs would surely profit from advancements in the interpretation of M_4,5_ edges HERFD XANES, which stands as the more direct probe of the 5f shell. Some advancements in this direction were recently demonstrated on bulk materials, using theoretical modeling of HERFD XANES and of the resonant inelastic X‐ray scattering.^[^ ^140^, ^141^, ^142^, ^143^ ^]^ Reaching a quantitative agreement between theory and experimental data would open the path to the interpretation of spectral differences observed in the HERFD XANES at M_4,5_‐edges of AnO_2_ NPs, comprehensive of changes in the local structure and in the oxidation state.

## Conflict of Interest

The authors declare no conflict of interest.
